# External Beam Radiation and Brachytherapy for Prostate Cancer: Is It a Possible Trigger of Large Cell Neuroendocrine Carcinoma of the Urinary Bladder?

**DOI:** 10.1155/2017/1853985

**Published:** 2017-05-30

**Authors:** Ali Zakaria, Bayan Al Share, Sri Kollepara, Cynthia Vakhariya

**Affiliations:** ^1^Department of Internal Medicine, Providence-Providence Park Hospital, Michigan State University College of Human Medicine, Southfield, MI, USA; ^2^Division of Hematology & Oncology, Providence-Providence Park Hospital, Michigan State University College of Human Medicine, Southfield, MI, USA

## Abstract

Neuroendocrine tumors commonly involve the respiratory and gastrointestinal systems. Primary genitourinary neuroendocrine tumors are rare, accounting for less than 1% of all bladder carcinomas. Four histopathologic subtypes have been described. Among those, large cell neuroendocrine carcinoma (LCNEC) is the least common, is more aggressive, and generally presents in an advanced stage with poor prognosis compared to transitional cell bladder carcinoma. There is no standardized treatment regimen because of the rarity of the disease. Herein, we present a case of 72-year-old male patient with previously treated prostate cancer, who received external beam radiation therapy and high dose brachytherapy, presenting with intermittent hematuria. Cystoscopy and transurethral resection of bladder tumor (TURBT) were performed. The histopathology and immunohistochemistry were consistent with large cell neuroendocrine carcinoma (LCNEC). Further studies are required to proof the higher risk of neuroendocrine carcinoma of the bladder in patients treated with external beam radiation therapy and brachytherapy for prostate cancer.

## 1. Introduction

Neuroendocrine carcinomas of the urinary bladder are rare. They account for less than 1% of all bladder carcinomas. Four histopathologic subtypes have been described. Among those, large cell neuroendocrine carcinoma (LCNEC) is the least common, is more aggressive, and generally presents in an advanced stage with poor prognosis compared to transitional cell bladder carcinoma. We are reporting this case to demonstrate that further studies are required to explore the association between neuroendocrine carcinoma of the bladder in patients treated with external beam radiation therapy and brachytherapy for prostate cancer.

## 2. Case Report

A 72-year-old male presented to the emergency department complaining of 6-week history of intermittent hematuria. He was diagnosed with prostate cancer thirteen years prior to this presentation and was treated with external beam radiation therapy. His cancer recurred six years later and he was treated with high dose brachytherapy. Five-year posttreatment follow-up with computed tomography (CT) scans and prostate specific antigen (PSA) levels (range 0.63–0.8 ng/mL) revealed no evidence of disease. He had a past medical history of coronary artery disease status after percutaneous intervention with drug-eluting stent to the right coronary artery, diabetes mellitus type 2, hypertension, obstructive sleep apnea, and complicated necrotizing pancreatitis with walled-off necrosis. He had a past surgical history of cardiac catheterization, distal pancreatectomy, and brachytherapy seeds implantation. He denied any previous history of smoking and alcohol or illicit drug use. He has a family history of diabetes and hypertension, but no history of malignancy. His home medications included aspirin, clopidogrel, atenolol, lisinopril, simvastatin, insulin, and as needed acetaminophen-oxycodone for pain. He has no known allergies. On physical examination he was alert, oriented, and not in acute distress. His vital signs were as follows: temp 36.4°C; blood pressure 134/75 mmHg; respiratory rate 16 bpm; pulse 116 bpm; and oxygen saturation 97% on room air. Abdominal examination revealed normal active bowel sounds, which was soft and nontender to palpation, no evidence of organomegaly, left-side nephrostomy tube with clear urine, and an indwelling urinary catheter with gross hematuria. Digital rectal exam revealed normal sphincter tone, with the prostate being smooth, symmetric, and measuring 4 × 5 cm. Fecal occult blood test (FOBT) was negative.

The patient presented to another hospital with same complaint of hematuria two months prior to this admission. At that time workup revealed left sided hydronephrosis due to obstructive ureteric stones. Urgent cystoscopy with ureteric stent placement was performed which failed to relieve the obstruction. So, an emergent nephrostomy tube was inserted and he was discharged with indwelling urinary catheter and follow-up plans for revision and placement of another anterograde ureteric stent.

**Figure 1:**
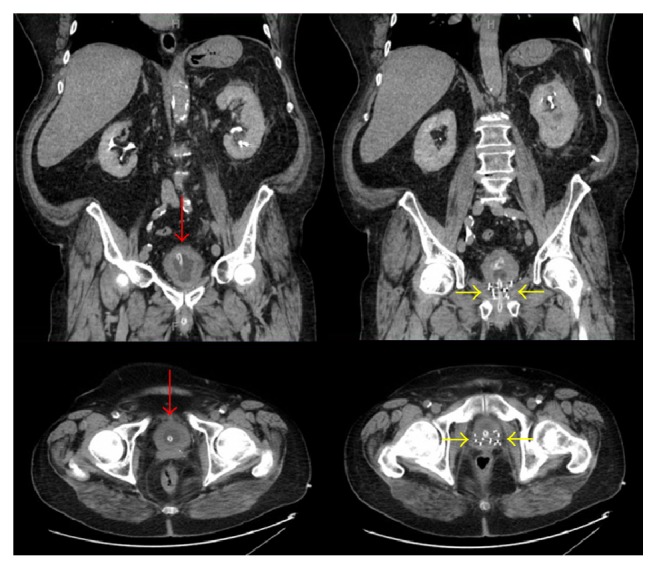
Computed tomography (CT) scan of the abdomen and pelvis revealed diffuse thickening of the wall of the urinary bladder with intravesical catheter* (red arrows)* and brachytherapy radiation seeds in the prostate* (yellow arrows)*.

**Figure 2:**
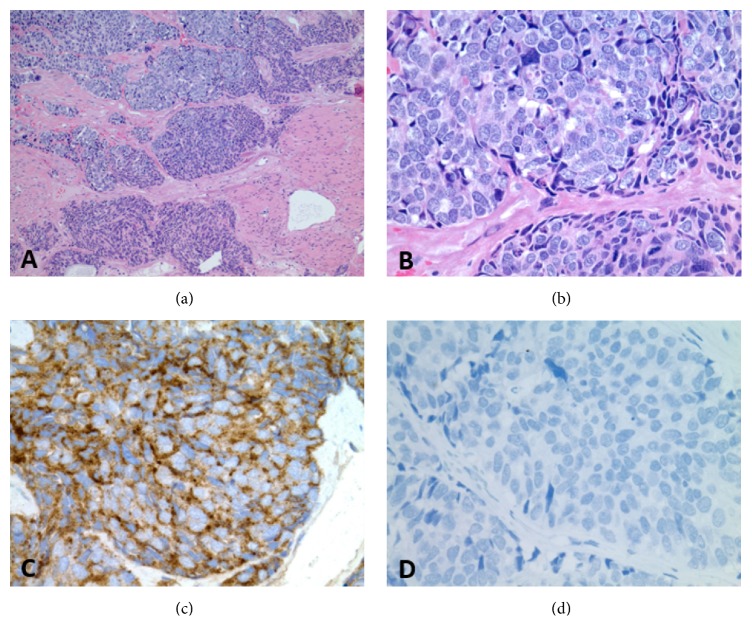
Histopathology of the resected bladder lesion. (a) Low-power field H&E stain reveals invasive poorly differentiated carcinoma infiltrates lamina propria and muscularis propria. (b) High-power field H&E stain reveals large polygonal-shape cells with low nuclear to cytoplasmic ratio, coarse chromatin structure, multiple nucleoli, and high rate of mitosis. (c) Immunohistochemical stain positive for chromogranin and (d) negative for PSA.

Upon admission to our hospital, the urinary catheter was replaced with a three-way urinary catheter and bladder irrigation was initiated. Computed tomography (CT) scan of abdomen and pelvis revealed no evidence of obstructive uropathy, diffuse thickening of the wall of the urinary bladder, and brachytherapy radiation seeds in the prostate (Figure 1). Cystoscopy revealed edematous mass over the left wall of the bladder. Transurethral resection of bladder tumor (TURBT) was performed and histopathology revealed invasive poorly differentiated carcinoma infiltrating lamina propria and muscularis propria, with foci suspicious for angiolymphatic invasion. Immunohistochemical stains were positive for Ber EP4, pankeratin, AE1/AE3, chromogranin, and TTF-1 and negative for CK-7, CK-20, PSA, and PAP (Figure 2). This carcinoma was histologically distinct from the previous prostate adenocarcinoma and consistent with large cell neuroendocrine carcinoma (LCNEC).

**Figure 3:**
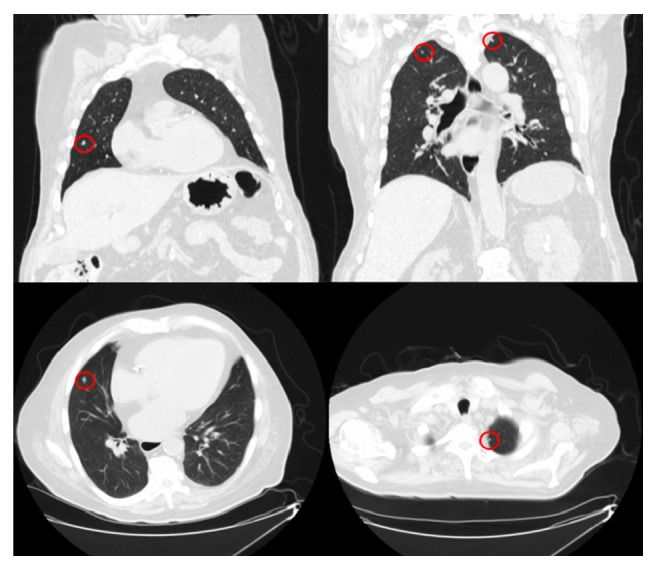
Computed tomography (CT) scan of the chest revealed multiple bilateral pulmonary and pleural-based nodules* (red circles)* suggestive of metastasis.

Further workup for staging of the disease was performed. Computed tomography (CT) scan of the chest revealed multiple bilateral pulmonary and pleural-based nodules suggestive of metastasis (Figure 3). Magnetic resonance imaging (MRI) of the brain and bone scan were both negative for metastatic disease.

Considering the rarity of the disease and unclear treatment guidelines a multidisciplinary team discussed the best treatment approach. The consensus was treatment with neoadjuvant chemotherapy using carboplatin and etoposide for five cycles followed by radical cystectomy. After completion of his fifth cycle of chemotherapy, a follow-up CT scan of the chest/abdomen/pelvis revealed interval increase in the size and number of bilateral pulmonary nodules and development of new liver lesions consistent with metastasis. He was started on anti-PD-1 immunotherapy with* pembrolizumab *“as an off-label prescription.” Nevertheless, he was readmitted to the hospital due to intractable shoulder and back pain. Palliative care team met with the patient and his family. He stated his wishes to be transferred to hospice with comfort care due to his significant declining performance status and quality of life.

## 3. Discussion

Neuroendocrine tumors commonly involve the respiratory and gastrointestinal systems. Primary genitourinary neuroendocrine tumors are rare, accounting for less than 1% of all bladder carcinomas [1, 2]. Four histopathologic subtypes have been described: small cell neuroendocrine carcinoma (SSNEC), large cell neuroendocrine carcinoma (LCNEC), carcinoid, and mixed histology. Literature review revealed the large cell subtype as the least common, with 25 cases reported as of 2013 [3]. It predominantly affects Caucasian men in seventh or eighth decade of life, with higher prevalence in patients with medical comorbidities, smoking history, and prior personal or family history of cancers [4–6]. However, the role of familial and genetic predisposition is still unknown. The etiology of bladder neuroendocrine tumor has not been clarified yet, with tumor development from stem cells or multipotent stem cells in urothelium being the widely accepted theory [7, 8]. In our case post-prostate-cancer external beam radiation therapy and high dose brachytherapy might be the trigger for this tumor.

The clinical presentation of LCNEC of the bladder may include abdominal pain, dysuria, obstructive voiding symptom, ureteral obstruction, or recurrent urinary tract infections (UTIs), yet painless hematuria is the most common presenting symptom [9, 10]. Given the rare incidence of the disease, treating physician should exclude other possible diagnoses such as metastasis of a pulmonary or gastrointestinal LCNEC, local invasion of the bladder by a poorly differentiated prostatic carcinoma, and poorly differentiated transitional cell carcinoma [8, 11, 12]. Tissue biopsy with characteristic histopathology and positive immunohistochemical markers demonstrating neuroendocrine differentiation (chromogranin, synaptophysin, neuron-specific enolase, and thyroid transcription factor-1) are required to establish and confirm the diagnosis of LCNEC [13–15].

Lung and liver have been reported as the most common sites for distant metastasis (and rarely the brain) in patients with LCNEC of the bladder either at time of diagnosis or later in the disease course [16]. Multiple imaging modalities have been suggested for staging of the disease, with computed tomography (CT) scan and/or magnetic resonance imaging (MRI) being most commonly used. In addition, positron emission tomography (PET) scan and octreoscan are other suggested modalities [17].

There is no standardized treatment regimen existing due to the rarity of the disease. Quek et al. reported that patients who received platinum based neoadjuvant chemotherapy using etoposide and cisplatin (or carboplatin) plus aggressive surgical approach with cystectomy and bilateral lymphadenectomy had significant better overall and recurrence-free survival than those treated with cystectomy alone [12]. Based on its rare incidence, prediction of overall survival and prognostic factors of LCNEC of the bladder is still difficult. However, overall survival in the reported cases ranged from <1 year for advanced disease to >2 years for early disease [8, 15, 17, 18]. Our patient had metastatic disease and his survival was 11 months after the initial diagnosis.

## 4. Conclusion

Large cell neuroendocrine carcinoma of the bladder is a very rare disease. Postradiation therapy for prostate cancer might be a risk factor for this disease as illustrated by our case. Unfortunately, there is no standardized treatment regimen so far, and the overall survival is poor.
